# Announcement

**DOI:** 10.1080/22221751.2018.1557846

**Published:** 2018-12-11

Based on the decision made at the Editorial Board meeting held on 12 October 2018, Emerging Microbe & Infections (EMI) will switch its publishing partner to Taylor and Francis effective from 1 January 2019.

But new submissions need to use new EMI website immediately. The new EMI website is https://www.tandfonline.com/temi.To directly access the submission site, please click https://mc.manuscriptcentral.com/temi

Since its launch in July 2012, EMI has worked closely with its previous publishing partner Nature Publishing Group (NPG, current name Springer Nature) and has seen its substantial growth during the last 6.5 years to become a major contributor to the global scientific fields of microbiology and infectious diseases with a special focus in emerging infections.

Nothing can be more revealing than the actual numbers:

- In 2012, EMI published 30 papers and the total paper to be published at the end of 2018 will be 210.
- EMI is now included in most key scientific indexes such as PubMed, Scopus, JCR, SCI among others. EMI received its first Impact Factor (IF) score of 2.258 in 2014 and its IF went up to 6.032 for 2017 based on the latest report.
- EMI is now ranked 15 out of 125 microbiology journals and 26 out of 155 immunology journals.
- At the same time, we have seen the submitted manuscripts have increased from 73 in 2013 to over 600 in 2018.

While it is happy to see the growth and the acceptance by our colleagues, EMI needs to do better to serve our authors and to make sure that our growth is sustainable. The large number of papers EMI needs to process each year has grown to a level that we can no longer afford to keep the original arrangement with the previous publishing partner.

EMI is extremely happy that Taylor & Francis (T&F) is wiling to be our future publishing partner by providing us with an attractive package including much enhanced customer service components. We are looking forward to a productive relationship with T&F.

EMI is also very grateful to the past and current colleagues at NPG/ SpringerNature. It is them who raised EMI from infancy to a future adulthood. EMI has been run by volunteer scientists thus any professional help from these professional colleagues was very valuable. We will always treasure this period of collaboration.

Any new manuscripts to EMI from now should be submitted via the following link: https://mc.manuscriptcentral.com/temi

For authors of manuscripts already submitted to EMI old site via www.nature.com/emi, you will be contacted with further instructions depending on the review status of your manuscripts.

Please contact EMI at editorial@emi2012.org with any questions, or check at the new EMI site https://www.tandfonline.com/temi for general information.

